# Supplementary material for: Benchmarking universal quantum gates via channel spectrum
Source: Nat Commun. 2023 Sep 21;14:5880. doi: 10.1038/s41467-023-41598-8 (PMC10514318; doi:10.1038/s41467-023-41598-8)
Supplement: Supplementary file 1 — Supplementary Information [file 41467_2023_41598_MOESM1_ESM.pdf]

Supplementary Information for
"Benchmarking universal quantum gates via channel spectrum"

Yanwu Gu,[1,2] Wei-Feng Zhuang,[1] Xudan Chai,[1,2] and Dong E. Liu[2,1,3,4]

[1]*Beijing Academy of Quantum Information Sciences, Beijing 100193, China*
[2]*State Key Laboratory of Low Dimensional Quantum Physics, Department of Physics, Tsinghua University, Beijing, 100084, China*
[3]*Frontier Science Center for Quantum Information, Beijing 100184, China*
[4]*Hefei National Laboratory, Hefei 230088, China*

(Dated: September 5, 2023)

In this supplementary, we provide some details about the central results in the main body of the paper.

- Supplementary Note 1 provides detailed proof of the relationship between noisy and ideal eigenvalues of the quantum channel by using the first-order perturbation theory.
- Supplementary Note 2 discusses the perturbation of channel eigenvalues under pure unitary error and the corresponding effect on our estimation of process fidelity.
- Supplementary Note 3 presents a randomized compiling procedure for benchmarking native gates using the symmetric group of the target gate as a twirling group.

Supplementary Note 1 – The relationship between noisy and ideal eigenvalues of quantum channels

In this section, we derive the relationship between the noisy channel eigenvalues of a gate and its corresponding ideal counterparts with the first order perturbation theory.

Consider a gate U acting on a d-dimensional space with eigen-decomposition $U|\phi_a\rangle = e^{i\lambda_a}|\phi_a\rangle$. As the actual implementation of a gate U is inevitably associated with some noise, it is more convenient to use quantum channels rather than quantum operators. Quantum channels are completely-positive trace-preserving (CPTP) maps, which transform one operator to another. The action of a quantum channel $\mathcal{E}$ on an arbitrary operator O can be characterized by a set of Kraus operators E_k, i.e., $\mathcal{E}(O) = \sum_k E_k O E_k^\dagger$. We denote the corresponding unitary channel of the unitary operator U as $\mathcal{U}$, whose action on an operator O is $\mathcal{U}(O) = UOU^\dagger$. Thus, the unitary channel $\mathcal{U}$ has the eigen-decomposition

$$\mathcal{U}(|\phi_a\rangle\langle\phi_b|) = U|\phi_a\rangle\langle\phi_b|U^\dagger = e^{i(\lambda_a-\lambda_b)}|\phi_a\rangle\langle\phi_b|. \tag{1}$$

Quantum channels are linear maps that can be represented as matrices under a set of the basis operators of the operator space, such as eigen-operators $|\phi_a\rangle\langle\phi_b|$. Meanwhile, operators are represented as vectors. The associated inner product between two operators A and B is the Hilbert-Schmidt inner product $\mathrm{tr}\{A^\dagger B\}$. Therefore, in this representation $\mathcal{U}$ is a unitary matrix.

Let us append a noise channel $\mathcal{E}$ to $\mathcal{U}$, with the noisy version of $\mathcal{U}$ denoted as $\widetilde{\mathcal{U}} = \mathcal{E}\mathcal{U}$. We investigate the relationship between the eigenvalues of $\widetilde{\mathcal{U}}$ and those of $\mathcal{U}$. If the noise is relatively weak, the problem is an eigenvalue perturbation of unitary matrix. Given the close relationship between unitary and Hermitian matrices, one can use Hermitian matrix perturbation theory to get the correction of eigenvalues and eigenstates, assuming a diagonalizable noisy gate $\widetilde{\mathcal{U}}$. In most cases, the assumption should be met in actual devices, since diagonalizable matrices are dense in the space of all matrices, meaning that any non-diagonalizable matrix can be deformed into a diagonalizable one by a small perturbation. In the following, we apply Hermitian perturbation theory to obtain the first order correction of the eigenvalues, and obtain the relationship between the noisy eigenvalues and ideal ones.

Define the eigenvalues and eigen-operators of $\widetilde{\mathcal{U}}$ as $g_{ab}e^{i\lambda_{ab}}$ and M_{ab}, that is

$$\widetilde{\mathcal{U}}(M_{ab}) = g_{ab}e^{i\lambda_{ab}}M_{ab}\,. \tag{2}$$

The perturbation matrix is

$$\Delta = \widetilde{\mathcal{U}} - \mathcal{U} = (\mathcal{E} - \mathcal{I})\,\mathcal{U}\,. \tag{3}$$

We assume the perturbation is small in terms of some matrix norm, such as the diamond norm $\|\Delta\|_\diamond = \delta$ [1]. Then, for a non-degenerate eigenvalue $e^{i(\lambda_a-\lambda_b)}$ with eigen-operator $|\phi_a\rangle\langle\phi_b|$, the first order correction is

$$\begin{aligned}\epsilon_1^{ab} &= \mathrm{tr}\big\{(|\phi_a\rangle\langle\phi_b|)^\dagger \Delta(|\phi_a\rangle\langle\phi_b|)\big\} \\ &= \mathrm{tr}\big\{(|\phi_a\rangle\langle\phi_b|)^\dagger (\mathcal{E}-\mathcal{I})\mathcal{U}(|\phi_a\rangle\langle\phi_b|)\big\} \\ &= e^{i(\lambda_a-\lambda_b)}\left[\mathrm{tr}\big\{(|\phi_a\rangle\langle\phi_b|)^\dagger \mathcal{E}(|\phi_a\rangle\langle\phi_b|)\big\}-1\right]. \end{aligned} \tag{4}$$

Thus, the noisy eigenvalue is approximated as

$$\begin{aligned} g_{ab}e^{i\lambda_{ab}} &\approx e^{i(\lambda_a-\lambda_b)}+\epsilon_1^{ab} \\ &= e^{i(\lambda_a-\lambda_b)}\mathrm{tr}\big\{(|\phi_a\rangle\langle\phi_b|)^\dagger \mathcal{E}(|\phi_a\rangle\langle\phi_b|)\big\}. \end{aligned} \tag{5}$$

In this case, the noisy eigen-operator M_{ab} is approximated by

$$M_{ab} \approx |\phi_a\rangle\langle\phi_b| + O(\delta) \tag{6}$$

where $O(\delta)$ are some correction terms with the first order of δ.

For degenerate eigenvalues $e^{i\lambda_n}$ with eigen-operators $|\phi_a\rangle\langle\phi_b|$ satisfying $\lambda_a-\lambda_b=\lambda_n$, these eigen-operators span a subspace. The $ab, a'b'$-entry of the perturbation matrix projected in this subspace is

$$\begin{aligned}\Delta^{(n)}_{ab,a'b'} &= \mathrm{tr}\big\{(|\phi_a\rangle\langle\phi_b|)^\dagger (\mathcal{E}-\mathcal{I})\mathcal{U}(|\phi_{a'}\rangle\langle\phi_{b'}|)\big\} \\ &= e^{i\lambda_n}\left[\mathrm{tr}\big\{(|\phi_a\rangle\langle\phi_b|)^\dagger \mathcal{E}(|\phi_{a'}\rangle\langle\phi_{b'}|)\big\}-\delta_{aa'}\delta_{bb'}\right] \\ &= e^{i\lambda_n}(\mathcal{E}^{(n)}_{ab,a'b'}-\mathcal{I}^{(n)}_{ab,a'b'}) \end{aligned} \tag{7}$$

where $\mathcal{E}^{(n)}_{ab,a'b'}$ and $\mathcal{I}^{(n)}_{ab,a'b'}$ are the entries of pure noise map $\mathcal{E}$ and Identity map $\mathcal{I}$ projected in this degenerate subspace. In the degenerate case, the first order corrections to the eigenvalue $e^{i\lambda_n}$ of $\mathcal{U}$ are the eigenvalues of the perturbation matrix $\Delta^{(n)}$. It's easy to find that the matrix $\Delta^{(n)}$ and the matrix $\mathcal{E}^{(n)}$ have the same eigen-operators M^0_{pq}, which are the superposition of eigen-operators $|\phi_a\rangle\langle\phi_b|$ in this degenerate subspace. They (M^0_{pq}) are also the corresponding unperturbed eigen-operators of noisy eigen-operator M_{pq} of $\widetilde{\mathcal{U}}$, that is

$$M_{pq} \approx M^0_{pq} + O(\delta)\,. \tag{8}$$

In this case, the eigenvalue $g_{pq}e^{i\lambda_{pq}}$ of $\widetilde{\mathcal{U}}$ is

$$\begin{aligned} g_{pq}e^{i\lambda_{pq}} &\approx e^{i\lambda_n} + \mathrm{tr}\Big\{G^{0\dagger}_{pq}\Delta^{(n)}(M^0_{pq})\Big\} \\ &= e^{i\lambda_n} + e^{i\lambda_n}\left[\mathrm{tr}\Big\{G^{0\dagger}_{pq}\mathcal{E}^{(n)}(M^0_{pq})\Big\}-1\right] \\ &= e^{i\lambda_n}\mathrm{tr}\Big\{G^{0\dagger}_{pq}\mathcal{E}^{(n)}(M^0_{pq})\Big\} \end{aligned} \tag{9}$$

where G^0_{pq} is the corresponding left eigen-operator of M^0_{pq} and they satisfy $\mathrm{tr}\big\{G^{0\dagger}_{pq}M^0_{p'q'}\big\}=\delta_{pq,p'q'}$. Therefore, the Eq. (9) has the same form as Eq. (5) but with a basis of a different form.

Supplementary Note 2 – Perturbation of channel eigenvalues under pure unitary error

Here we consider the noisy eigenvalues of a quantum gate under a pure unitary error

$$V = e^{-iH_e\delta} \tag{10}$$

where H_e is the Hamiltonian of error and δ characterize the error strength. Assume the target gate $U=e^{-iH\theta}$. Thus the operator of noisy gate is $\widetilde{U}=VU$. In this case, the process fidelity is

$$\begin{aligned} F &= \frac{|\mathrm{tr}\{V\}|^2}{d^2} \\ &\approx \frac{|\mathrm{tr}\big\{I - iH_e\delta - \frac{1}{2}H_e^2)\delta^2\big\}|^2}{d^2} \\ &= \frac{d^2 - d\,\mathrm{tr}\big\{H_e^2\big\}\delta^2}{d^2} \\ &= 1-\frac{\mathrm{tr}\big\{H_e^2\big\}}{d}\delta^2 \end{aligned} \tag{11}$$

where we use the property $\text{tr}\{H_e\} = 0$ and keep the term up to $O(\delta^2)$. This is a well-known result that unitary error with some matrix norm δ has process infidelity of order $O(\delta^2)$ [1].

We then analyze how eigenvalues of a quantum channel $\mathcal{U}$ change under such unitary error with perturbation theory. Now the effect of the noisy gate $\widetilde{\mathcal{U}}$ on an operator O is $\widetilde{\mathcal{U}}(O) = VUOU^\dagger V^\dagger$. Represent all quantum channels as matrices, the perturbation matrix is

$$\Delta = \widetilde{\mathcal{U}} - \mathcal{U}\,. \tag{12}$$

For a non-degenerate eigen-operator $|\phi_a\rangle\langle\phi_b|$ with eigenvalue $e^{i(\lambda_a-\lambda_b)}$, the first order correction is

$$\begin{aligned}\epsilon_1^{ab} &= \text{tr}\big\{(|\phi_a\rangle\langle\phi_b|)^\dagger \Delta(|\phi_a\rangle\langle\phi_b|)\big\}\\ &= e^{i(\lambda_a-\lambda_b)}\left[\text{tr}\big\{(|\phi_a\rangle\langle\phi_b|)^\dagger V|\phi_a\rangle\langle\phi_b|V^\dagger\big\} - 1\right].\end{aligned} \tag{13}$$

To further expand this equation, we will use the Baker-Hausdorff (BH) lemma

$$e^X Y e^{-X} = e^{\text{ad}_X}(Y) = \sum_{n=0}^{\infty}\frac{\text{ad}_X^n(Y)}{n!} \tag{14}$$

where ad is a map on operators with the effect $\text{ad}_X(Y) = [X, Y]$. Then the first order correction is

$$\begin{aligned}\epsilon_1^{ab} &= e^{i(\lambda_a-\lambda_b)}\left[\text{tr}\big\{(|\phi_a\rangle\langle\phi_b|)^\dagger e^{-i\delta\text{ad}_{H_e}}(|\phi_a\rangle\langle\phi_b|)\big\} - 1\right]\\ &\approx e^{i(\lambda_a-\lambda_b)}\left[\text{tr}\left\{(|\phi_a\rangle\langle\phi_b|)^\dagger\left(\mathcal{I} - i\delta\text{ad}_{H_e} - \frac{1}{2}\delta^2\text{ad}_{H_e}^2\right)(|\phi_a\rangle\langle\phi_b|)\right\} - 1\right]\\ &= e^{i(\lambda_a-\lambda_b)}\left[\underbrace{-i\delta\left(\langle\phi_a|H_e|\phi_a\rangle - \langle\phi_b|H_e|\phi_b\rangle\right)}_{\epsilon_{1,1}^{ab}}\underbrace{-\frac{1}{2}\delta^2\text{tr}\big\{(|\phi_a\rangle\langle\phi_b|)^\dagger\text{ad}_{H_e}^2(|\phi_a\rangle\langle\phi_b|)\big\}}_{\epsilon_{1,2}^{ab}}\right].\end{aligned} \tag{15}$$

If we consider only the first-order correction, the noisy eigenvalue is

$$g_{ab}e^{i\lambda_{ab}} \approx e^{i(\lambda_a-\lambda_b)} + \epsilon_1^{ab} = e^{i(\lambda_a-\lambda_b)}(1 + \epsilon_{1,1}^{ab} + \epsilon_{1,2}^{ab}). \tag{16}$$

Then, we get the estimate of process fidelity

$$\begin{aligned}\hat{F} &= \frac{1}{d^2}\sum_{ab}\mathcal{E}_{ab,ab}\\ &\approx \frac{1}{d^2}\sum_{ab} g_{ab}e^{i\lambda_{ab}}e^{-i(\lambda_a-\lambda_b)}\\ &= 1 + \frac{1}{d^2}\sum_{ab}\epsilon_{1,1}^{ab} + \frac{1}{d^2}\sum_{ab}\epsilon_{1,2}^{ab}\end{aligned} \tag{17}$$

where the term with order $O(\delta)$ is

$$\begin{aligned}&\frac{1}{d^2}\sum_{ab}\epsilon_{1,1}^{ab}\\ &= \frac{1}{d^2}\sum_{ab} -i\delta\left(\langle\phi_a|H_e|\phi_a\rangle - \langle\phi_b|H_e|\phi_b\rangle\right)\\ &= 0\end{aligned} \tag{18}$$

and the term with order $O(\delta^2)$ is

$$\begin{aligned}
&\frac{1}{d^2}\sum_{ab}\epsilon_{1,2}^{ab}\\
=&-\frac{\delta^2}{2d^2}\sum_{ab}\mathrm{tr}\big\{(|\phi_a\rangle\langle\phi_b|)^\dagger[H_e,[H_e,|\phi_a\rangle\langle\phi_b|]]\big\}\\
=&-\frac{\delta^2}{2d^2}\sum_{ab}\langle\phi_a|H_e^2|\phi_a\rangle-2\langle\phi_a|H_e|\phi_a\rangle\langle\phi_b|H_e|\phi_b\rangle+\langle\phi_b|H_e^2|\phi_b\rangle\\
=&-\frac{\delta^2}{2d^2}(2d\mathrm{tr}\big\{H_e^2\big\}-2\mathrm{tr}\{H_e\}^2)\\
=&-\frac{1}{d}\mathrm{tr}\big\{H_e^2\big\}\delta^2\,. \qquad (19)
\end{aligned}$$

This coincides with the expression in Eq. (11).

However, due to the first order correction only contributing a term with order $O(\delta^2)$, we must also take into account the second order correction to the eigenvalues. The second order correction is

$$\begin{aligned}
\epsilon_2^{ab} &= \sum_{mn\neq ab}\frac{|\mathrm{tr}\big\{(|\phi_m\rangle\langle\phi_n|)^\dagger\Delta(|\phi_a\rangle\langle\phi_b|)\big\}|^2}{e^{i(\lambda_a-\lambda_b)}-e^{i(\lambda_m-\lambda_n)}}\\
&= \sum_{mn\neq ab}\frac{|\mathrm{tr}\big\{(|\phi_m\rangle\langle\phi_n|)^\dagger V|\phi_a\rangle\langle\phi_b|V^\dagger\big\}|^2}{e^{i(\lambda_a-\lambda_b)}-e^{i(\lambda_m-\lambda_n)}}\\
&= \sum_{mn\neq ab}\frac{|\langle\phi_m|V|\phi_a\rangle|^2|\langle\phi_n|V|\phi_b\rangle|^2}{e^{i(\lambda_a-\lambda_b)}-e^{i(\lambda_m-\lambda_n)}}\\
&\approx \sum_{mn\neq ab}\frac{\big(\delta_{am}+(\langle\phi_m|H_e|\phi_a\rangle\langle\phi_a|H_e|\phi_m\rangle-\langle\phi_m|H_e^2|\phi_a\rangle\delta_{am})\delta^2\big)\big(\delta_{bn}+(\langle\phi_n|H_e|\phi_b\rangle\langle\phi_b|H_e|\phi_n\rangle-\langle\phi_n|H_e^2|\phi_b\rangle\delta_{bn})\delta^2\big)}{e^{i(\lambda_a-\lambda_b)}-e^{i(\lambda_m-\lambda_n)}}\\
&= \sum_{m\neq a}\frac{\langle\phi_m|H_e|\phi_a\rangle\langle\phi_a|H_e|\phi_m\rangle\delta^2}{e^{i(\lambda_a-\lambda_b)}-e^{i(\lambda_m-\lambda_b)}}+\sum_{n\neq b}\frac{\langle\phi_n|H_e|\phi_b\rangle\langle\phi_b|H_e|\phi_n\rangle\delta^2}{e^{i(\lambda_a-\lambda_b)}-e^{i(\lambda_a-\lambda_n)}}\,. \qquad (20)
\end{aligned}$$

If the error Hamiltonian H_e is diagonal under the basis of eigenvectors of U, the second order correction is $\epsilon_2^{ab}=0$ up to the second order $O(\delta^2)$. There is no problem with our method.

However, except in the special case, there is some discrepancy between the process fidelity estimated using our method and the actual value, due to the presence of the term ϵ_2^{ab} in the noisy eigenvalue $g_{ab}e^{i\lambda_{ab}}$.

Here, we can directly compute the noisy eigenvalues of the channel $\widetilde{\mathcal{U}}$ from the eigenvalues of the operator $\widetilde{U}$ and give the analytical form of estimated process fidelity by our method. We first compute the Hamiltonian of $\widetilde{U}$ by Baker-Campbell-Hausdorff formula

$$\begin{aligned}
H'=\log(VU) &= \log(e^{-iH_e\delta}e^{-iH\theta})\\
&\approx -iH\theta-iH_e\delta-\frac{1}{2}\mathrm{ad}_{-iH\theta}(-iH_e\delta)+\frac{1}{12}\mathrm{ad}^2_{-iH\theta}(-iH_e\delta)-\frac{1}{720}\mathrm{ad}^4_{-iH\theta}(-iH_e\delta)+\cdots \qquad (21)
\end{aligned}$$

where we only keep the terms up to the order $O(\delta)$ and omit some terms with the ad map. We can compute the eigenvalues of H' compared to those of the $-iH\theta$ with the first order perturbation theory. The first order correction to the eigenvalue $i\lambda_a$ with eigenstate $|\phi_a\rangle$ is

$$\begin{aligned}
\epsilon_1^a &= \langle\phi_a|H'-(-iH\theta)|\phi_a\rangle\\
&= -i\delta\langle\phi_a|H_e|\phi_a\rangle \qquad (22)
\end{aligned}$$

where these terms with ad map are all zeros because

$$\begin{aligned}
&\langle\phi_a|\mathrm{ad}_{-iH\theta}(O)|\phi_a\rangle\\
=&-i\theta\langle\phi_a|(HO-OH)|\phi_a\rangle\\
=&\ i\lambda_a(\langle\phi_a|O|\phi_a\rangle-\langle\phi_a|O|\phi_a\rangle)=0 \qquad (23)
\end{aligned}$$

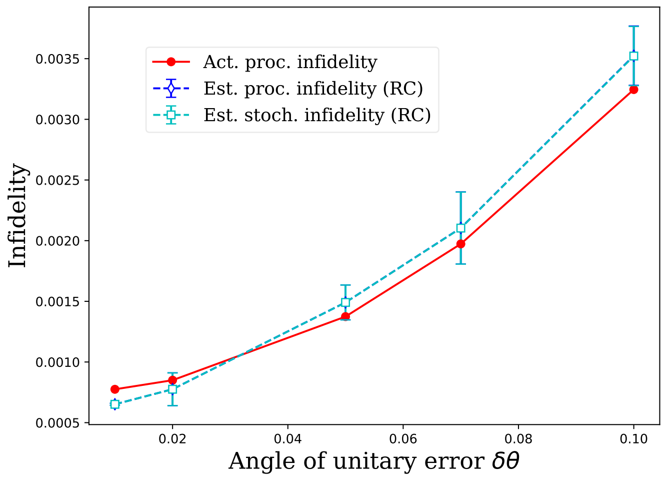

FIG. 1: Benchmarking of T gate with randomized compiling. In simulation, stochastic error is fixed ($\delta p = 0.001$) and unitary is $R_X(\delta\theta)$ with varied error angle $\delta\theta$. The twirling group is $\mathbf{T} = \{I, Z\}$. For each original circuit, we generate $N_r = 10$ random circuits and each random circuit is run for $N_s = 10^3$ times.

where O is any operator. Then the noisy eigenvalue of $|\phi_a\rangle\langle\phi_b|$ is

$$g_{ab}e^{i\lambda_{ab}} \approx e^{i\lambda_a + \epsilon_1^a - i\lambda_b - \epsilon_1^b} . \tag{24}$$

Thus our estimator for process fidelity is

$$\begin{aligned} \hat{F} &= \frac{1}{d^2}\sum_{ab} e^{\epsilon_1^a - \epsilon_1^b} \\ &= 1 - \frac{\sum_a \langle\phi_a|H_e|\phi_a\rangle^2}{d}\delta^2 . \end{aligned} \tag{25}$$

Because the term $\sum_a \langle\phi_a|H_e|\phi_a\rangle^2$ is always smaller than the term $\mathrm{tr}\{H_e^2\}$ except when H_e is a diagonal matrix under the basis $|\phi_a\rangle$, our method under-estimates process infidelity under unitary error in general. This problem can be fixed by introducing some randomization procedure into the benchmarking circuits to convert unitary errors to stochastic errors [2–4].

Supplementary Note 3 – Randomized compiling with the symmetric group of the target gate

For a circuit composed of single-qubit and two-qubit gates, randomized compiling (RC) is a standard procedure to tailor the noise into stochastic Pauli noise with Pauli twirling. Here, we consider another case that the circuit is the repetition of a native gate U, that is U^L. In the spirit of RC, if considering U as hard gate, we need a twirling group $\mathbf{T}$, whose element T_i should be transformed to another T_j under the conjugate operation of U, that is $UT_iU^\dagger = T_j$.

A simple example of this type of group is a symmetric group (or centralizer) of U

$$\mathbf{T} = \{T : U^\dagger T U = T\} . \tag{26}$$

For the sequence of U^L, random gates from the twirling group $\mathbf{T}$ are introduced before each application of U, but the effect of these random gates should be canceled before the next U is applied. Finally, we get a new random sequence

$$T_L^\dagger U(T_L T_{L-1}^\dagger)\cdots U(T_i T_{i-1}^\dagger)\cdots U(T_2 T_1^\dagger) U T_1 \tag{27}$$

where the gates in parentheses should be implemented as one gate. In actual implementation, all the gates should be associated with noise, and the gate sequence is denoted as the composition of quantum channels

$$\begin{aligned} & \mathcal{T}_L^\dagger \mathcal{E}_T \mathcal{E}\mathcal{U}\mathcal{T}_L \mathcal{T}_{L-1}^\dagger \mathcal{E}_T \cdots \mathcal{E}\mathcal{U}\mathcal{T}_2\mathcal{T}_1^\dagger \mathcal{E}_T \mathcal{E}\mathcal{U}\mathcal{T}_1\mathcal{E}_T \\ = \; & \mathcal{T}_L^\dagger \mathcal{E}_T \mathcal{E}\mathcal{T}_L \mathcal{U}\mathcal{T}_{L-1}^\dagger \mathcal{E}_T \cdots \mathcal{E}\mathcal{T}_2\mathcal{U}\mathcal{T}_1^\dagger \mathcal{E}_T \mathcal{E}\mathcal{T}_1\mathcal{U}\mathcal{E}_T \end{aligned} \tag{28}$$

where we use the property that the gates in group $\mathbf{T}$ commute with U. We assume the noise of each twirling gate is the same quantum channel $\mathcal{E}_T$ for simplicity (the noisy twirling gates are $\mathcal{T}\mathcal{E}_T$), but this assumption can be relaxed [3].

After averaging many such random sequences we get

$$\left(\frac{1}{N_T}\sum_{\mathcal{T}_L}\mathcal{T}_L^\dagger\mathcal{E}_T\mathcal{E}\mathcal{T}_L\right)\mathcal{U}\cdots\left(\frac{1}{N_T}\sum_{\mathcal{T}_1}\mathcal{T}_1^\dagger\mathcal{E}_T\mathcal{E}\mathcal{T}_1\right)\mathcal{U}\mathcal{E}_T \tag{29}$$

where N_T is the number of gates in the twirling group $\mathbf{T}$. The effect of this randomization procedure is to transform the noise to stochastic noise, that is applying a random quantum channel $\mathcal{T}^\dagger\mathcal{E}_T\mathcal{E}\mathcal{T}$ with probability $\frac{1}{N_T}$. One can use group representation theory to get a simpler form of noise. But in our case, this subtlety is not necessary. This procedure is similar to the Ref. [2]. However, we do not require the twirling group to be abelian and do not need the assumption that there is no equal irreducible representation for a symmetric group. Thus our method has high flexibility to choose a twirling group.

We use a simulated experiment to show the performance of this procedure. We benchmark T gate under a unitary error $R_X(\delta\theta)$ with varied error angle $\delta\theta$ and fixed stochastic error $\delta p = 0.001$. We choose $\mathbf{T} = \{I, Z\}$ as twirling group. For each original circuit, we generate $N_r = 10$ random circuits and each random circuit is run for $N_s = 10^3$ times.

The theory in Supplementary Note 2 shows that the process infidelity estimated by our method is of the order $O(\delta\theta^4)$ without the use of randomized compiling (similar effect that measured (experimental) error rate is very different from the actual (model) error rate is also observed in randomized benchmarking [5–7]). However, by introducing randomized compiling, our method can accurately estimate the process infidelity, as demonstrated in Supplementary Figure 1. It is important to note that the process infidelity measured using randomized compiling on the native gate includes the noise from both the target gate and the twirling gates since the twirling gates are not merged into the original circuit in the same way as when using randomized compiling on circuit fragments. For simplicity, we did not add noise to the twirling gates in this case. To obtain the infidelity of the target gate alone, it is necessary to benchmark the twirling gates separately and subtract their contribution from the overall infidelity, similar to the process used in interleaved RB [8]. However, for our CSB there is greater flexibility to choose the twirling group as we do not need the good properties of group twirling as in the interleaved RB and the target gate does not need to be in the twirling group. So we can choose a group of gates whose error is much smaller than the target gate such that the extra error introduced by twirling gates can be neglected.

[1] M. Kliesch and I. Roth, PRX Quantum **2**, 010201 (2021).
[2] E. Onorati, A. H. Werner, and J. Eisert, Phys. Rev. Lett. **123**, 060501 (2019).
[3] J. J. Wallman and J. Emerson, Phys. Rev. A **94**, 052325 (2016).
[4] A. Hashim, R. K. Naik, A. Morvan, J.-L. Ville, B. Mitchell, J. M. Kreikebaum, M. Davis, E. Smith, C. Iancu, K. P. O'Brien, I. Hincks, J. J. Wallman, J. Emerson, and I. Siddiqi, Phys. Rev. X **11**, 041039 (2021).
[5] T. Proctor, K. Rudinger, K. Young, M. Sarovar, and R. Blume-Kohout, Phys. Rev. Lett. **119**, 130502 (2017).
[6] J. J. Wallman, Quantum **2**, 47 (2018).
[7] J. Qi and H. K. Ng, International Journal of Quantum Information **17**, 1950031 (2019).
[8] A. Carignan-Dugas, J. J. Wallman, and J. Emerson, New Journal of Physics **21**, 053016 (2019).
